# Area-Consistent Aggregation of SDGSAT-1 Nighttime Light Data: A Radiant Flux Framework for Cross-City Population Estimation

**DOI:** 10.3390/s26175664

**Published:** 2026-09-06

**Authors:** Jinke Liu, Yifei Zhu, Xuesheng Zhao, Wenbin Sun, Fan Yang

**Affiliations:** 1 College of Geoscience and Surveying Engineering, China University of Mining and Technology-Beijing, Beijing 100083, China; liujinkers@gmail.com (J.L.); zxs@cumtb.edu.cn (X.Z.); swb@cumtb.edu.cn (W.S.); 2 State Key Laboratory for Tunnel Engineering, China University of Mining and Technology-Beijing, Beijing 100083, China; venuszyf0603@163.com; 3 Information Center of Ministry of Natural Resources, Beijing 100036, China

**Keywords:** NTL remote sensing, SDGSAT-1, radiant flux, area-consistent aggregation, radiance

## Abstract

**Highlights:**

**What are the main findings?**

- Converting SDGSAT-1 NTL from radiance to physical radiant flux enables area-consistent regional aggregation.
- Flux-based SDGSAT-1 nighttime light data improve cross-city population estimation compared with radiance-based aggregation.

**What are the implications of the main findings?**

- Converting SDGSAT-1 NTL from radiance to physical radiant flux accounts for latitude-related grid-area variation during regional aggregation of gridded SDGSAT-1 data.
- Using flux-based SDGSAT-1 nighttime light data can improve cross-city population estimation.

**Abstract:**

Nighttime light (NTL) data is widely used to monitor urban development, economic activity and population distribution, supporting socio-economic analysis and sustainable development planning. SDGSAT-1 NTL data, with its multiple bands and high resolution, has become an important source for studying human activity patterns. However, directly summing radiance values on geographic grids assumes equal contributions from all grid cells, regardless of the differences in the ground area represented by each cell. As a result, regional nighttime light totals may be systematically distorted, particularly in cross-latitude analyses where pixel areas differ substantially. To address this aggregation issue, this study proposes a physically explicit area-weighting framework that converts SDGSAT-1 radiance to radiant flux by incorporating the actual surface area represented by each grid cell. The framework is validated using 25 cities spanning different latitudes and development levels in the Northern Hemisphere. Results show that the radiant flux model, which captures total emitted power, demonstrates improved performance over radiance-based aggregation (a density-based measure) in population estimation (R^2^ = 0.85 vs. 0.82). By shifting from light intensity to integrated total energy, this approach improves cross-latitude comparability and enhances the methodological robustness of NTL-based analyses.

## 1. Introduction

Over the past century, the diffusion of artificial lighting has effectively extended the functional day of cities, enabling work, mobility, and consumption to persist after dark and thereby supporting broad socio-economic development [1,2,3]. Nighttime light (NTL) remote sensing has become an important tool for monitoring human activity at night, as it provides valuable data on the intensity and spatial patterns of nighttime light [4,5]. Satellite sensors capture NTL data that records the brightness distribution on Earth’s surface, which is widely used to estimate socio-economic indicators [6,7], monitor urbanization [8,9], analyze spatial dynamics [10,11], and assess public emergencies [12,13], all of which support policy-making and sustainable development [14,15].

NTL data is widely used to assess light pollution and socio-economic conditions by examining its spatial distribution and radiance variations [16,17,18]. Radiance, defined as the radiant flux per unit projected area per unit solid angle, is a physically standardized and correct quantity for characterizing the local intensity of light emanating from a grid cell [19]. It is the appropriate measure for grid-level or local analyses. However, a common practice in large-scale socio-economic studies is to aggregate these grid-level radiances by summation to represent the total light output of a city or region [20,21]. It is at this stage of aggregation for regional totals that a systematic bias is introduced. This bias stems from two interconnected issues rooted in the Earth’s sphericity. First, the ground area represented by a single grid cell varies significantly with latitude. A grid cell at a high latitude (e.g., in Stockholm) covers a considerably smaller surface area than a grid cell at a low latitude (e.g., in Singapore). Summing radiance values directly, therefore, conflates the intensity of light with an unequal spatial sampling footprint, unfairly weighting high-latitude grids and making cross-latitude comparisons of total light unreliable. Second, and consequently, the sum of radiances lacks clear physical meaning; it is not a measure of total emitted energy. Adding a density-based quantity (radiance) across unequal areas does not yield a well-defined physical quantity like total power or radiant flux. These issues together create an underestimation of total light output at higher latitudes, complicating the separation of true activity intensity from geographic scale effects and ultimately reducing the robustness of NTL-based cross-city comparisons.

Given these issues, directly using radiance as a proxy for regional total light output may affect the physical consistency and cross-latitude comparability of NTL-derived indicators in applications such as socio-economic estimation, urban development assessment, and light pollution analysis. This concern has attracted considerable academic attention. For instance, Elvidge and others innovatively adjusted grid values to compensate for the effects of grid area changes in DMSP-OLS data, developing a calibrated dataset, the DMSP-OLS Calibration Data. The core of this method is directly multiplying the grid area as a coefficient to correct the systemic biases caused by area differences [22,23]. Similarly, Kyba et al. adjusted grid values for VIIRS/DNB data to compensate for the effects of grid area variation, aiming to eliminate the system errors caused by area differences and more accurately assess the radiance and area changes in NTL [24]. Furthermore, Zeng and others further considered the errors introduced by latitude differences when using radiance summation to measure energy intensity and proposed a corresponding calibration strategy for VIIRS/DNB data. This approach enhances the cross-latitude comparability and physical consistency of VIIRS/DNB-derived metrics [25].

However, it is worth noting that the calibration methods mentioned above primarily focus on DMSP/OLS and VIIRS/DNB data. Although these datasets have significant advantages, such as long time series and global coverage, they also have clear limitations, such as single spectral bands and relatively coarse spatial resolutions (DMSP/OLS at 1000 m, NPP/VIIRS at 500 m) [26,27,28]. These limitations have affected the performance of NTL data in fine-scale applications. In recent years, with continuous advancements in remote sensing technology, the SDGSAT-1 NTL data, successfully launched in 2021 and available for free access, has brought new developments in NTL research [29,30,31]. SDGSAT-1 data can record NTL information with high spatial resolution and multi-band recording capabilities [32,33]. The spatial resolution of the panchromatic and color NTL data reaches 10 m and 40 m, respectively, marking a breakthrough in color NTL detection and providing a finer and richer information source for NTL studies [34,35,36]. In the future, SDGSAT-1 data will undoubtedly exhibit great potential for applications in socio-economic assessments and light pollution research [37,38,39]. However, the current lack of correction strategies for latitude-induced biases in SDGSAT-1 NTL data remains a key issue that needs to be addressed. Solving this problem will further enhance the cross-latitude comparability and physical consistency of NTL observations, thereby providing more robust data support for related fields.

To address the bias introduced by using a density-based metric (radiance) to represent regional NTL totals across different latitudes, and to ensure the physical interpretability of cross-latitude comparisons, this study introduces radiant flux (a measure of total emitted power) as the appropriate metric for aggregated NTL data. This approach explicitly corrects for grid area variations, thereby transforming the analysis from aggregating local intensity to quantifying total energy output. The core contributions of this research are as follows: First, we propose a physically grounded conversion framework for SDGSAT-1 NTL observations that converts radiance to radiant flux by explicitly incorporating each grid’s true surface area. This design eliminates latitude-driven grid-area biases and enables fair, projection-robust regional aggregation and cross-latitude comparison with clear physical interpretability. Second, to validate the credibility and effectiveness of the proposed method, we selected 25 representative cities from different latitudes, regions, and development levels in the Northern Hemisphere as research samples, and established a fitting relationship with population data to evaluate the effectiveness of radiant flux in practical applications, while also comparing the changes in NTL data before and after applying the conversion model.

## 2. Materials and Methods

### 2.1. Study Area

**Figure 1:**
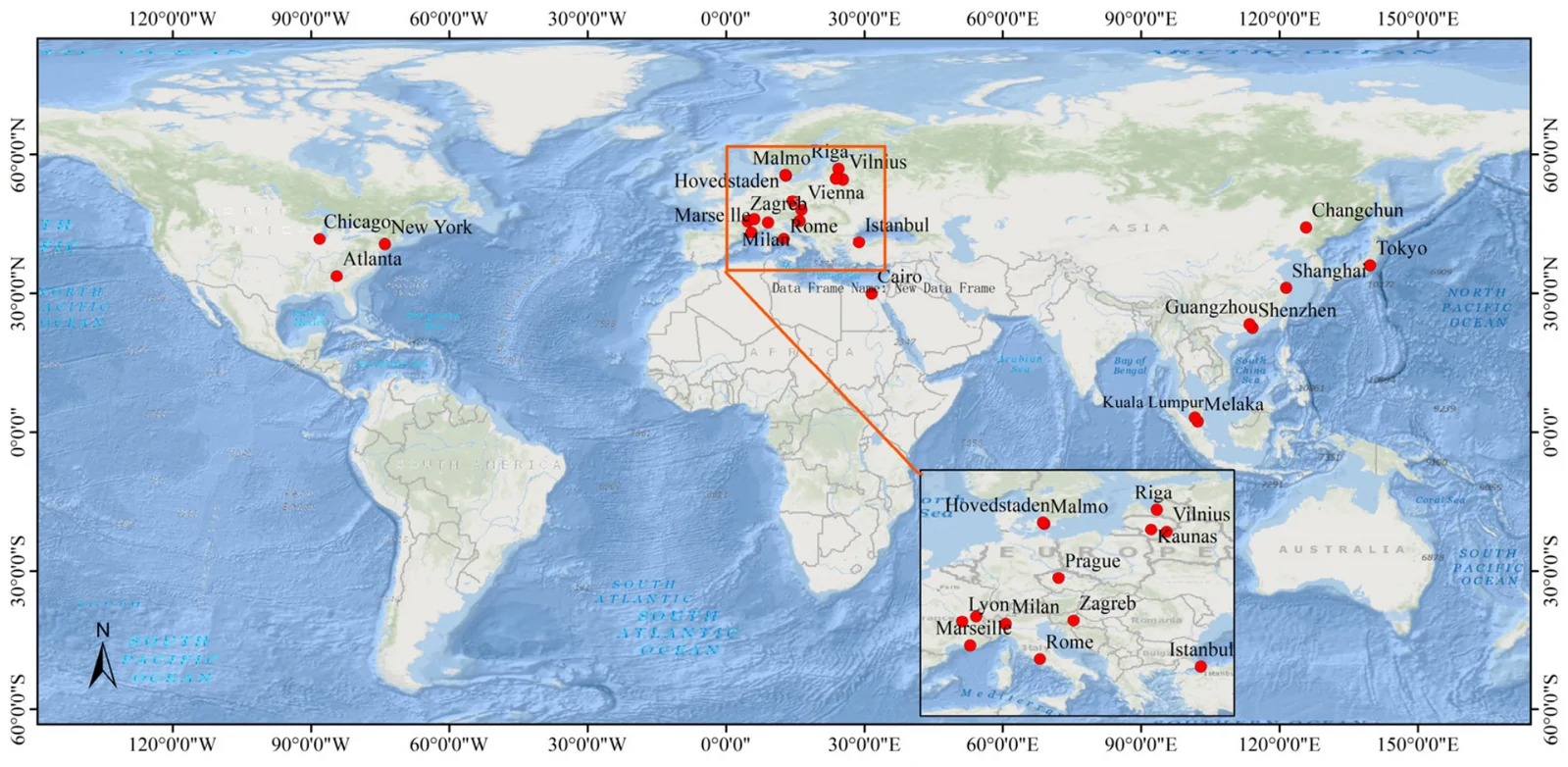
Spatial distribution of 25 representative cities worldwide.

**Figure 2:**
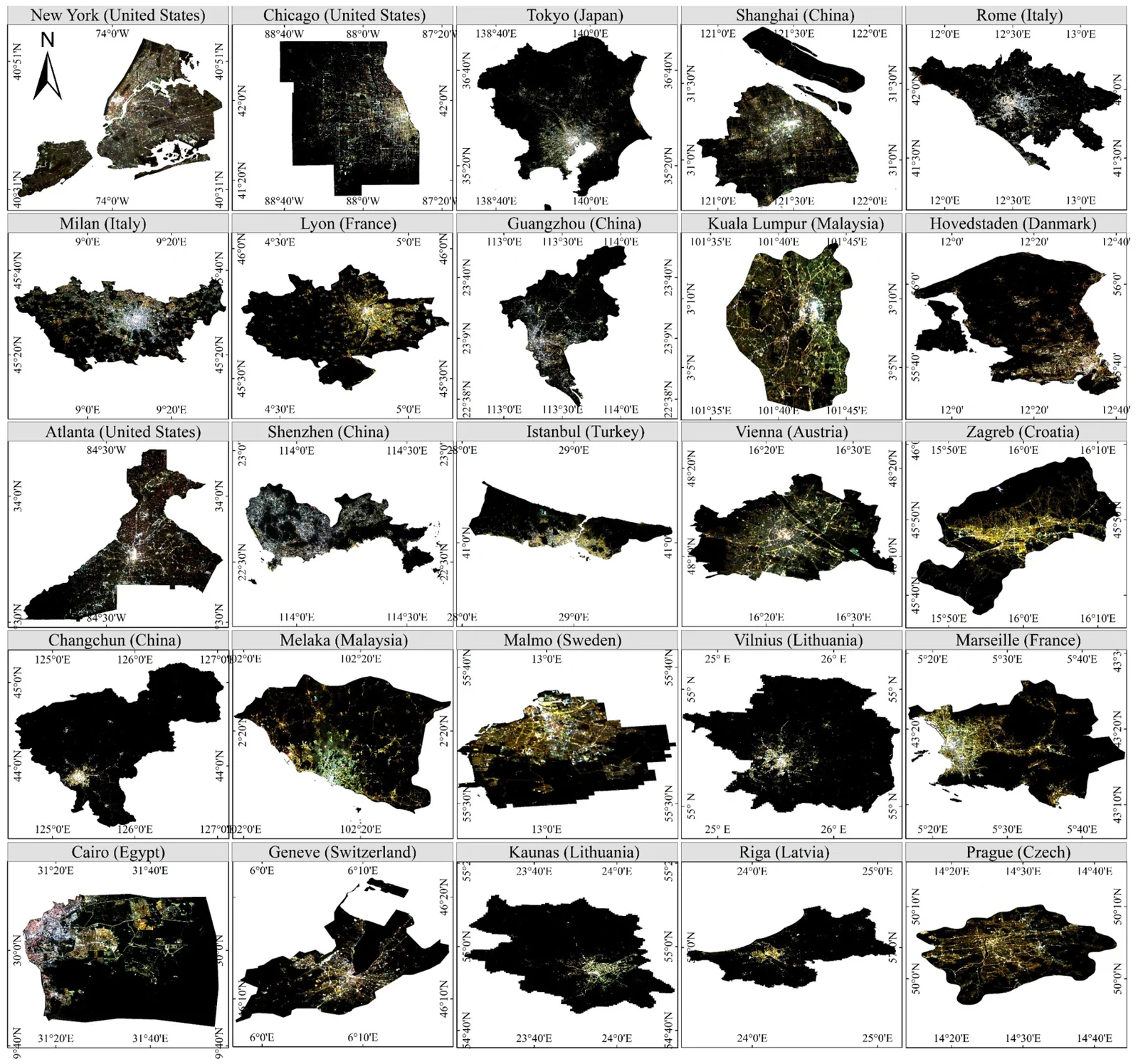
SDGSAT-1 imagery of 25 representative cities in the Northern Hemisphere with different latitudes and levels of development.

To systematically analyze the impact of latitude effects on nighttime radiance and radiant flux, this study selected 25 representative cities in the Northern Hemisphere as research samples. These cities span across different latitudinal zones, are distributed across multiple continents, and include both developed economies and developing regions (Figure 1 and Figure 2). Considering that the seasonal reversal phenomenon between the Northern and Southern Hemispheres may cause additional biases in NTL observations, and the poor coverage of SDGSAT data (especially in Africa and South America), this study limits the analysis to cities in the Northern Hemisphere. This sampling strategy helps reduce potential seasonal differences among the selected cities and limits the influence of inter-hemispheric seasonal variation on the analysis, thereby improving the comparability and interpretability of the results. At the same time, the Northern Hemisphere offers a rich set of urban samples and a broad latitudinal gradient, providing ideal conditions for latitude sensitivity analysis.

Regarding spatial distribution, the sample in this study covers a complete gradient from low to high latitudes: from tropical cities near the equator—such as Malacca (2° N) and Kuala Lumpur (3° N) in Malaysia—to mid- and high-latitude European cities—such as Hovedstaden (56° N) in Denmark and Riga (57° N) in Lithuania—forming a latitude span of approximately 55°, sufficient to reveal the systematic impacts of the earth’s curvature and grid area variation on NTL radiant flux. Geographically, the sample cities are widely distributed across major economic and cultural regions of the world, including North America: New York, Chicago, and Atlanta, representing typical high-energy consumption, densely illuminated developed cities; Europe, including Milan, Vienna, Lyon, and Copenhagen, characterized by both historical urban areas and modern industrial zones; Asia, including Tokyo, Shanghai, Shenzhen, and Kuala Lumpur, reflecting the rapid urbanization model of East and Southeast Asian cities; and North Africa, including Cairo, representing the NTL characteristics of densely populated cities in arid climates.

### 2.2. Data Collection

To support SDG monitoring, the Chinese Academy of Sciences launched SDGSAT-1 in 2021, a satellite designed to provide comprehensive, high-precision data for the observation of human-environment interactions. SDGSAT-1 is a pioneering satellite in the field of global NTL monitoring, offering a significant advancement in tracking environmental and socio-economic changes, especially in urban areas. Equipped with the Glimmer Imager (GLI), a sensor optimized for NTL detection, it provides unprecedented accuracy in capturing the intensity and distribution of artificial light across the Earth’s surface [40,41]. The GLI provides nighttime light observations at two spatial resolutions: 10 m for the panchromatic band and 40 m for the RGB bands. Although the panchromatic band provides a finer spatial resolution, its higher noise level and greater preprocessing complexity make it less suitable for stable quantitative radiometric analysis. In contrast, the RGB bands provide more stable radiometric measurements and are better suited to the subsequent radiometric calibration, band integration, and radiant-flux calculation [42]. These advancements significantly improve upon earlier satellite systems such as DMSP/OLS and VIIRS, making SDGSAT-1 invaluable for studying urban populations, energy consumption, light pollution, and SDG indicators. The satellite’s ability to monitor these dimensions across vast urban regions is critical for assessing global sustainability trends and implementing effective policies. This study uses SDGSAT-1 data from 25 cities in the Northern Hemisphere, downloaded in March 2022, supplemented with 2023 data for missing areas. Geometric misalignment varies across different bands. Although the panchromatic band provides a higher spatial resolution (10 m), its noise characteristics require a more complex denoising and correction procedure. In contrast, the RGB bands (40 m resolution) exhibit lower noise levels and provide more stable radiometric measurements. Since this study focuses on regional NTL radiant flux estimation rather than fine-scale grid-level change detection, the RGB bands were selected to ensure greater consistency and stability in the subsequent analysis.

To avoid potential uncertainties introduced by spatial downscaling and boundary-based aggregation of gridded population products, city-level population data were collected directly from the official statistical agencies of the corresponding countries. Specifically, we used officially reported population totals for each city rather than deriving population estimates by aggregating existing gridded population datasets within administrative boundaries. In addition, the reference year of the population data was matched to the acquisition year of the corresponding SDGSAT-1 nighttime light imagery. Population statistics for 2022 were used for cities represented by SDGSAT-1 observations acquired in 2022, whereas 2023 population statistics were used for cities supplemented with imagery acquired in 2023. This temporal matching reduces potential bias arising from interannual changes in both population size and nighttime light intensity. For each city, the geographical boundary used for nighttime light aggregation was matched to the administrative or statistical unit represented by the corresponding census population data.

To enhance the comparability of NTL information across different sensors and to verify the robustness of the radiant flux framework under different data sources, this study also incorporates NTL data from the VIIRS (Visible Infrared Imaging Radiometer Suite) Day/Night Band (DNB) onboard the Suomi-NPP/NOAA-20 satellite (https://www.class.noaa.gov/ (accessed on 15 March 2026)). VIIRS/DNB is one of the most widely used NTL remote sensing products worldwide, offering advantages such as stable imaging, long time series, and global coverage. Since 2012, it has continuously provided high-quality nighttime radiant observations [43,44]. Its spatial resolution is about 500 m, effectively capturing the light distribution characteristics of urban clusters, large transportation corridors, and industrial facilities, making it an important data source for monitoring large-scale human activity and energy consumption. VIIRS/DNB uses wide-field scanning imaging and enhances low-brightness signals in the visible to near-infrared range (about 500–900 nm) to achieve radiometric measurements in weak light environments, including moonlight illumination, water reflections, fishing boat lights, combustion fire points, and urban lighting. This product has good temporal continuity, avoiding issues of radiometric inconsistencies across years and satellites, and is widely used in socio-economic studies, including population, GDP, electricity consumption, urban expansion, and disaster assessment.

## 3. Methods

**Figure 3:**
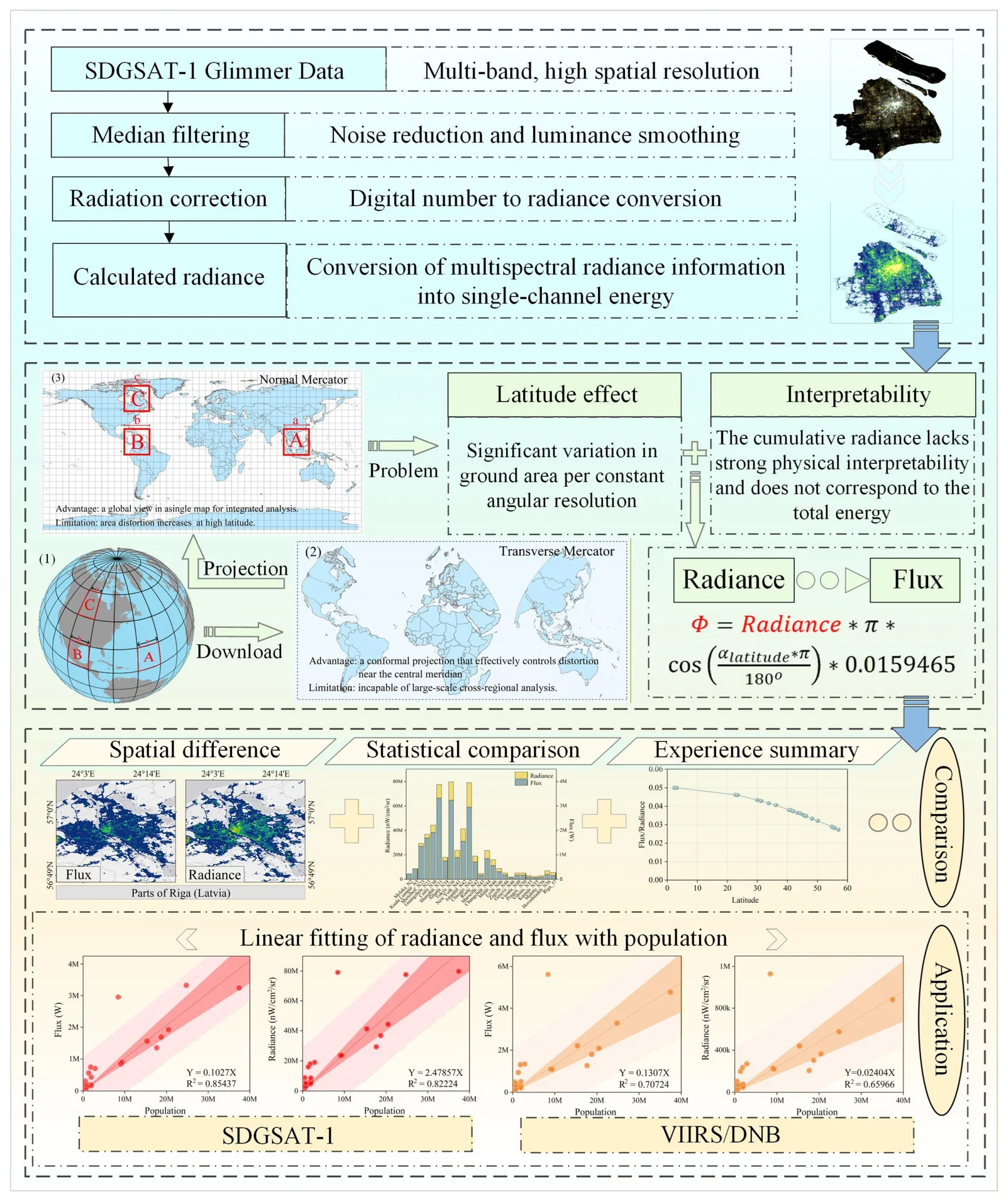
Research flowchart for converting radiance to radiant flux of SDGSAT-1 data.

The main work of this study includes three stages: (1) Data acquisition and preprocessing: 25 representative cities from different latitudes in the Northern Hemisphere were selected, and cloud-free, geometrically accurate RGB band data were extracted from SDGSAT-1 NTL imagery. Median filtering, radiometric calibration, and grayscale brightness conversion were performed to ensure the physical consistency of radiant signals. (2) Model construction and theoretical derivation: Based on the Earth’s ellipsoidal geometry, we derived a latitude-dependent function to compute each grid’s true surface area. Direct aggregation of gridded SDGSAT-1 Level-4 radiance under a non-equal-area spatial framework may introduce latitude-related grid-area effects. (3) Results validation and application analysis: We separately regressed population against radiant flux and radiance across the 25-city sample, and compared their predictive performance. Additionally, the impact of resolution changes and data source differences on the stability of the results was explored (Figure 3). Collectively, these steps establish a framework spanning theoretical derivation, preprocessing, flux conversion, and validation, enabling latitude-consistent estimation of nighttime radiant energy and supporting cross-latitude, energy-based applications of SDGSAT-1 NTL data within the Northern Hemisphere.

### 3.1. Theoretical Foundation

The SDGSAT-1 nighttime light data used in this study are provided as Level-4 products in the Transverse Mercator projection. As a conformal projection, the Transverse Mercator projection effectively controls geometric distortion near the central meridian and preserves local shape, making it suitable for fine-scale spatial analysis and urban- or regional-level mapping. Rather than reprocessing lower-level unprojected sensor data, we used the publicly available Level-4 product as the starting point because it has already undergone the standard geometric and radiometric processing required for quantitative analysis, thereby providing a consistent and computationally straightforward basis for subsequent calibration and spatial aggregation. The imagery was then transformed to a unified WGS 84-based spatial reference system to facilitate spatial overlay and comparison among the selected cities and other geospatial datasets. Because the spatial framework used for subsequent aggregation is not equal-area, the ground area represented by individual grid cells varies systematically with latitude. Therefore, grid-cell area must be explicitly considered when regional nighttime light totals are calculated.

It is important to distinguish the imaging geometry of the SDGSAT-1 Glimmer Imager from the grid-area variation present in the gridded Level-4 product. The GLI acquires nighttime observations using a push-broom imaging mechanism and records the received signal initially as digital numbers, which are subsequently calibrated to radiance. Radiance is a physically valid intensive quantity describing radiant power per unit projected area and per unit solid angle. Variations in the instantaneous ground footprint of the sensor are primarily associated with scanning and viewing geometry, particularly the across-track position of the observed target, rather than being caused directly by the latitude of the target area. The issue addressed in this study therefore does not represent an intrinsic latitude-dependent radiometric bias in the GLI observations. Instead, it arises when gridded radiance values are directly aggregated within a geographic or other non-equal-area spatial framework.

**Figure 4:**
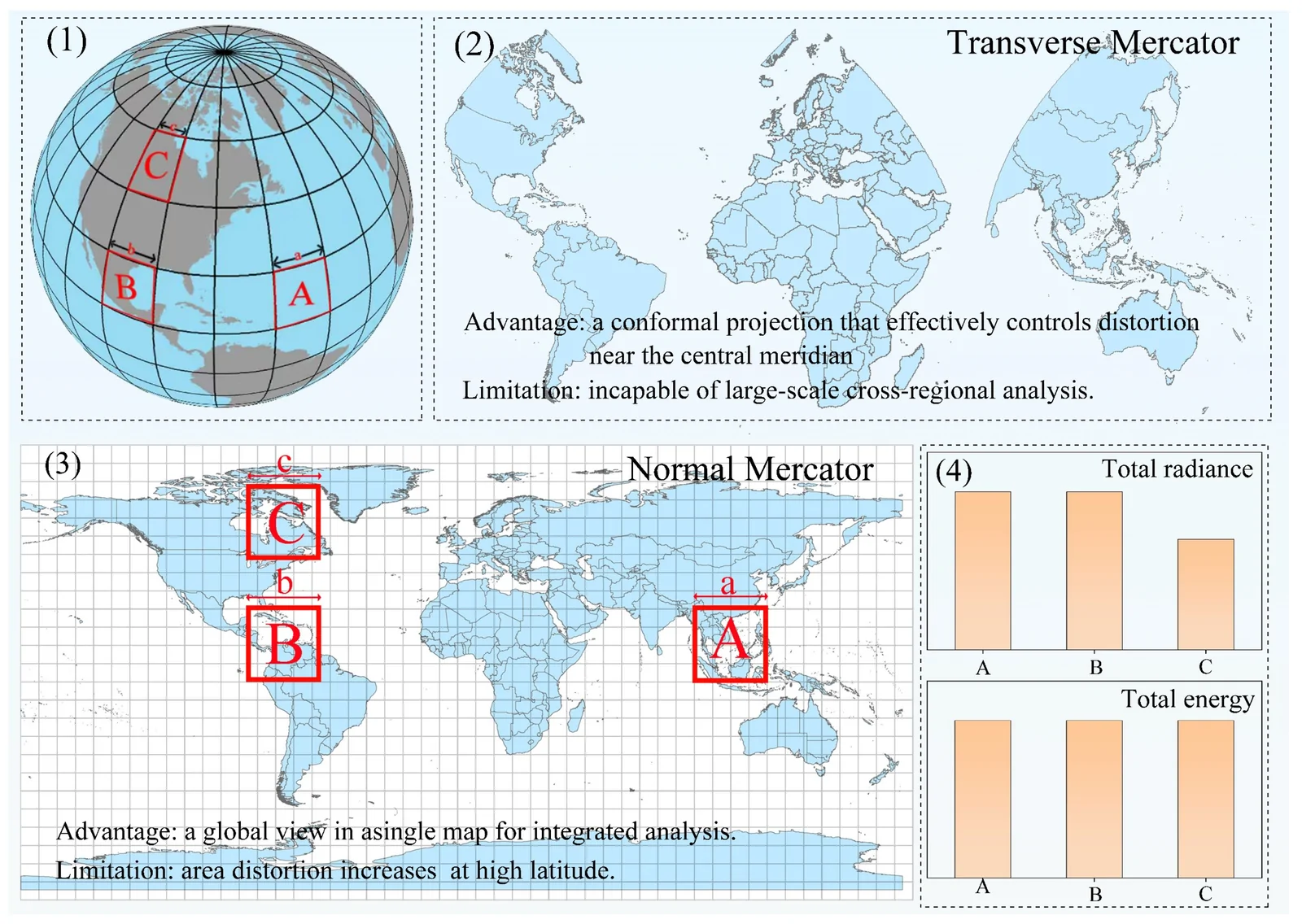
Theoretical analysis of the reasons for latitude differences in NTL data.

In previous nighttime light studies, grid-level radiance values have often been summed to characterize the overall nighttime light level of a city or region. Although this procedure is computationally straightforward, the direct sum of radiance does not explicitly account for differences in the ground area represented by individual grids. Under a non-equal-area grid, grids at different latitudes may cover unequal surface areas, and direct summation consequently assigns equal numerical weight to spatial footprints of different sizes. To illustrate this issue, an idealized geometric model is presented in Figure 4. Regions A, B, and C contain the same number of grid cells, with A and B located at lower latitudes and C located at a higher latitude. Because the length of a parallel decreases with increasing latitude, the cells in region C represent a smaller total surface area than those in regions A and B. Accordingly, even when the mean radiance and grid count are identical among the three regions, their actual area-integrated light outputs are not equivalent. Directly summing radiance would produce identical totals and would therefore overestimate the area-integrated light output of the higher-latitude region relative to the lower-latitude regions.

The radiant-flux framework proposed in this study represents an alternative and physically explicit implementation of area weighting. Radiance (W·m^−2^·sr^−1^) remains the appropriate quantity for characterizing local light intensity, identifying individual light sources, mapping urban structures, and comparing grid-level brightness. Under the adopted Lambertian approximation, each grid’s radiance is multiplied by π and by the actual surface area represented by that grid to obtain an area-integrated radiant-flux estimate. The grid-level flux values can then be summed within a defined spatial domain, such as a city or administrative unit, to characterize its total nighttime light output. Therefore, the proposed method does not modify or correct the original radiance measurements themselves. Rather, it converts an intensive radiometric quantity into an area-integrated extensive quantity during regional aggregation. Its purpose is to improve the physical interpretability of regional nighttime light totals and the consistency of cross-latitude comparisons under non-equal-area spatial frameworks. In the present study, this framework is evaluated primarily through cross-city population estimation in the Northern Hemisphere. Its applicability to other socioeconomic indicators, ecological assessments, and Southern Hemisphere regions requires further independent validation.

### 3.2. SDGSAT-1 NTL Data Preprocessing

In general, SDGSAT-1 NTL data exhibits significant multi-band characteristics, with each band independently recording the radiance information of surface grid cells within different spectral ranges at night. Specifically, the red (R), green (G), and blue (B) bands correspond to different spectral ranges and can reflect the emission characteristics of different types of artificial light sources (such as LEDs, sodium lamps, mercury lamps, etc.). A comprehensive analysis of these bands can not only reveal the spectral differences in urban lighting structures but also provide a basis for light pollution classification and energy consumption estimation. In lit areas, the radiance of grid cells is usually much higher than the set minimum threshold due to the strong influence of artificial lighting, exhibiting clear high-brightness features. In non-lit areas or natural surfaces (such as water bodies or forests), the grid brightness values are often close to the minimum due to the lack of light sources, resulting in dark areas in the imagery. However, due to factors such as sensor noise, cosmic ray interference, atmospheric scattering, and surface reflectance heterogeneity, NTL images often contain abnormal grid cells, which exhibit inconsistent characteristics across different bands [45]. For example, they may show unusually low or high brightness in one band, while maintaining normal values in other bands, thereby disrupting spectral consistency between bands. If not processed, these abnormal grid cells can cause systematic interference in image radiometric consistency, flux calculation, and subsequent modeling analysis. To address this, the study introduces a median filtering method during the data preprocessing stage to suppress noise and smooth the brightness of SDGSAT-1 NTL imagery.

After completing noise suppression and quality optimization for the original SDGSAT-1 NTL imagery, this study further performs radiometric calibration on its three RGB bands to convert digital numbers (DN) into physically meaningful radiance values. This process aims to eliminate systematic biases caused by sensor characteristics, detection gain, offset errors, and differences in imaging conditions, allowing the image to reflect the actual nighttime lighting level on the Earth’s surface more accurately. The original DN values of SDGSAT-1 NTL images only represent the relative signal strength received by the satellite detector and lack direct physical significance. Since the detection sensitivity, optical system transmittance, and radiant response curves vary across different bands, without calibration, it would be impossible to maintain radiometric consistency across bands, temporal phases, and regions. Therefore, radiometric calibration is a key step in the physical quantification of NTL remote sensing data and serves as the foundational process for building subsequent radiant flux models. In this study, the radiometric calibration process considers various factors, including the satellite sensor’s gain and bias parameters, atmospheric transmittance, and surface reflectance characteristics. By applying the DN values to the calibration equations provided by SDGSAT-1’s official documentation, precise conversion from digital quantized values to radiance (unit: W/m^2^/sr/μm) is achieved. The calibration formula is as follows:

(1) L=DN×Gain+Bias

where L refers to the radiance of the image, measured in watts per square meter per steradian per micrometer (W/m^2^/sr/μm). Gain is a calibration parameter that adjusts the sensor’s sensitivity to light. Bias represents a constant offset or systematic error introduced by the sensor’s measurement system. The relevant parameters are shown in Table 1.

Subsequently, to facilitate comparison with VIIRS/DNB data, the radiant units of the GLI band were converted to the commonly used brightness unit nW/cm^2^/sr.

(2) $$L_{convert}=L\times W\times 10^{5}$$

where $L_{convert}$ is the radiance of the SDGSAT-1 NTL data after conversion, and **W** is the bandwidth of the band. The bandwidths of the R, G, and B bands of the SDGSAT-1 NTL image are 294 nm, 106 nm, and 102 nm, respectively. Since the calibrated radiance is expressed in W/m^2^/sr/μm, the spectral bandwidths were converted from nanometers to micrometers before radiometric integration. Therefore, the effective bandwidths used in the calculation were 0.294 μm, 0.106 μm, and 0.102 μm for the R, G, and B bands, respectively.

To further quantify the integrated radiant intensity of the RGB three bands, this study integrates the radiant brightness of each band to calculate the grayscale brightness, which represents the overall light energy level. Grayscale brightness is a commonly used method for converting multispectral radiant information into a single-channel energy indicator. It simplifies the spectral dimension while preserving the overall brightness characteristics, facilitating subsequent radiant flux calculations and spatial statistical analysis. This study adopts the grayscale conversion method for color images [46]. The method not only effectively retains the brightness distribution and structural details of the original image, but also maintains robust performance under varying lighting conditions, color temperature differences, and noisy environments. The grayscale brightness value of SDGSAT-1 NTL is calculated using the following formula:

(3) $$Brightness=0.2989\times C_{R}+0.5870\times C_{G}+0.1140\times C_{B}$$

where $C_{R}$,$C_{G}$, $C_{B}$ represent the radiance of the red, green, and blue bands of the SDGSAT-1 NTL data, respectively.

### 3.3. Conversion of Radiance to Radiant Flux

Under the assumption of isotropic emission within the observed hemisphere, the relationship between radiance and radiant flux can be expressed as follows:

(4) $$Radiance=\frac{d^{2}\Phi }{d\Omega dA\cos\theta }$$

The definition of the radiance of SDGSAT-1 data is as follows: Where Ω is the solid angle, dAcosθ is the projected area in the observation direction, and Φ is the radiant flux. The expression for radiant flux is:

(5) Φ=∫∫Radiance∗cosθ dΩ dA

where Φ is the radiant flux, and Ω is the solid angle.

In previous NTL remote sensing studies, scholars have widely focused on the anisotropic characteristics of nighttime radiance, especially in applications based on VIIRS/DNB data. Several studies have shown that nighttime radiance is not uniformly distributed in an ideal sense, but is influenced by multiple factors, such as the type of light sources, surface geometry, observation angles, and atmospheric scattering [47,48,49]. However, to date, there has been no systematic study of anisotropy for SDGSAT-1 NTL imagery. This is mainly due to the unique characteristics of the SDGSAT-1 nighttime imaging system—its observation geometry, optical system design, and RGB multi-band structure differ from previous single-band NTL sensors. On the other hand, the complexity of NTL sources further increases the modeling difficulty: ground lighting is typically composed of multiple directional light sources (such as street lamps, billboards, and vehicle lights), whose emission directions, intensity distribution, and occlusion relationships are highly complex and are significantly affected by building shading, terrain slope, and surface reflectivity variations. Additionally, atmospheric scattering and secondary reflection effects lead to highly nonlinear characteristics of the energy received by the sensor under different incident angles. Accurately characterizing this three-dimensional radiant transfer process on a global scale still faces multiple challenges in terms of data, models, and computational capabilities [50,51].

For the reasons mentioned above, this study does not include anisotropic factors in the radiant flux calculation framework at the current stage. Instead, it adopts a simplified assumption: it assumes that the radiant behavior of NTL grid cells follows the isotropy principle, meaning that the radiant energy of each grid cell is emitted uniformly in all directions within the hemisphere, regardless of the observation angle or surface geometry. This assumption is a common idealized approximation in radiant transfer theory, and particularly in the absence of multi-angle observation conditions, it significantly reduces model complexity while maintaining energy conservation and directional averaging in a statistically reasonable manner.

By adopting this assumption, the radiant flux calculation formula can be simplified from the complete directional integral form to an area integral model under isotropic conditions, making it easier to implement unified energy estimation and latitude correction analysis on the Northern Hemisphere scale. Although this treatment theoretically omits local directional features, its error, compared to the grid area correction term, is negligible at macro scales (e.g., urban agglomerations, national regions, or latitude bands), thus ensuring high applicability and computational stability. The expression for radiant flux can be represented as:

(6) Φ=Radiance∗S∗π

where Φ is the radiant flux, and S is the grid area.

It should be noted that urban nighttime light emissions are not perfectly Lambertian due to anisotropic emission from artificial light sources, three-dimensional urban structures, and viewing geometry effects. Therefore, the calculated radiant flux represents an area-integrated radiant-flux estimate under the adopted assumption rather than the absolute physical upward emission.

### 3.4. Derivation of Grid Area Conversion

In the GCS_WGS_1984 coordinate reference system, the ellipsoid model used has an equatorial radius of approximately 6378.137 km and a polar radius of approximately 6356.752 km. These two parameters define the shape and size of the Earth. Based on these parameters, we can calculate the length of the Earth’s equator using the circumference formula, which is approximately 40,075 km ($l_{0}$), the longest circumference on the Earth’s surface. Meanwhile, the meridian length of the ellipsoid, the arc connecting the south and north poles and perpendicular to the equator, is approximately 39,941 km ($l_{m}$).

The resolution of the SDGSAT data in the GCS_WGS_1984 coordinate system can be calculated using the following formula:

(7) $$r=\frac{0.04*360}{40075}=0.000359326$$

The length of other parallels parallel to the equator can be calculated using the following formula:

(8) $$l_{\alpha }=\cos\left(\frac{\alpha_{latitude}*\pi }{180^{◦}}\right)*l_{0}$$

where $\alpha_{laitude}$ is the latitude, $l_{\alpha }$ is the length of the parallel at latitude $\alpha_{laitude}$, and $l_{0}$ is the length of the equator.

The length of the grid centerline parallel to the latitude line is denoted as m, while the height parallel to the longitude line is denoted as h, which does not change with latitude.

(9) $$m=\frac{l_{\alpha }*0.04}{40075}=\cos\left(\frac{\alpha_{latitude}*\pi }{180^{◦}}\right)*0.04$$

(10) $$h=\frac{l_{m}*r}{360}\approx 0.03986625$$

where $l_{m}$ is the meridian length of the ellipsoid, and r is the angular resolution.

The area of the grid is:

(11) $$S=m*h=\cos\left(\frac{\alpha_{latitude}*\pi }{180^{◦}}\right)*0.04*\frac{l_{m}*r}{360}\approx \cos\left(\frac{\alpha_{latitude}*\pi }{180^{◦}}\right)*0.00159465$$

**Figure 5:**
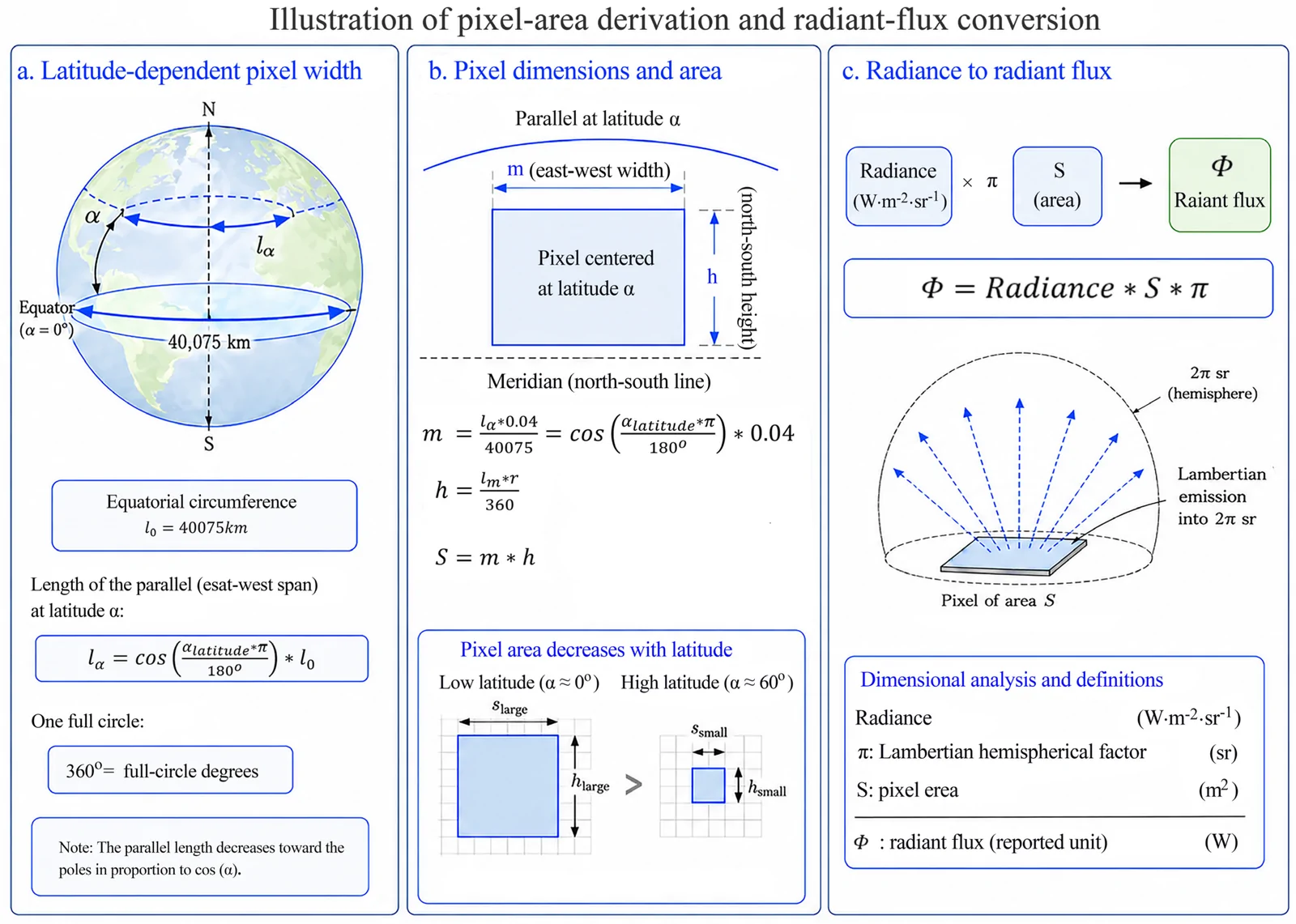
Illustration of pixel-area derivation and radiant-flux conversion.

It is worth noting that the unit of radiance is nW/cm^2^/sr, while the unit of radiant flux is W. To convert it to radiant emittance, we multiply by π, and then multiply by $S_{p}$ to convert it to radiant flux. However, the unit of $S_{p}$ is km^2^, so the calculated radiant flux was initially expressed at a 10 W scale. To standardize the numerical representation and obtain the final radiant flux values in watts, a conversion factor of 10 was applied. Therefore, the final radiant flux is expressed in W.

(12) $$\Phi =Radiance*\pi *\cos\left(\frac{\alpha_{latitude}*\pi }{180^{◦}}\right)*0.00159465*10$$

where Radiance is the radiance (in units of nW/cm^2^/sr), and Φ is the radiant flux (in units of W). By applying this formula, we can independently calculate the radiant flux for each grid in the SDGSAT data product, thereby more accurately revealing the energy intensity distribution of NTL across different geographic regions, particularly in different latitude bands (Figure 5).

## 4. Results

### 4.1. Radiant Flux vs. Radiance for Population Estimation

In past research, the depiction of NTL has generally relied on the single dimension of total radiance. While this method is intuitive, it has a significant limitation: when radiance is aggregated at the grid level, its original meaning, which accurately describes the physical phenomenon, is often lost. Additionally, the ground area represented by each grid is not constant across latitudes. Grid cells at higher latitudes often correspond to smaller ground areas than those at lower latitudes, which can bias grid-wise aggregation and undermine cross-regional comparisons based on summed radiance. In light of this, our study adopts radiant flux to measure NTL grid cells. The physical meaning of radiant flux lies in its description of the transfer of radiant energy over time. It is a purely objective physical quantity with the unit of power, typically measured in watts. This shift in perspective means that we are not only focusing on the intensity of individual grid brightness but also on how these intensities accumulate spatially.

**Figure 6:**
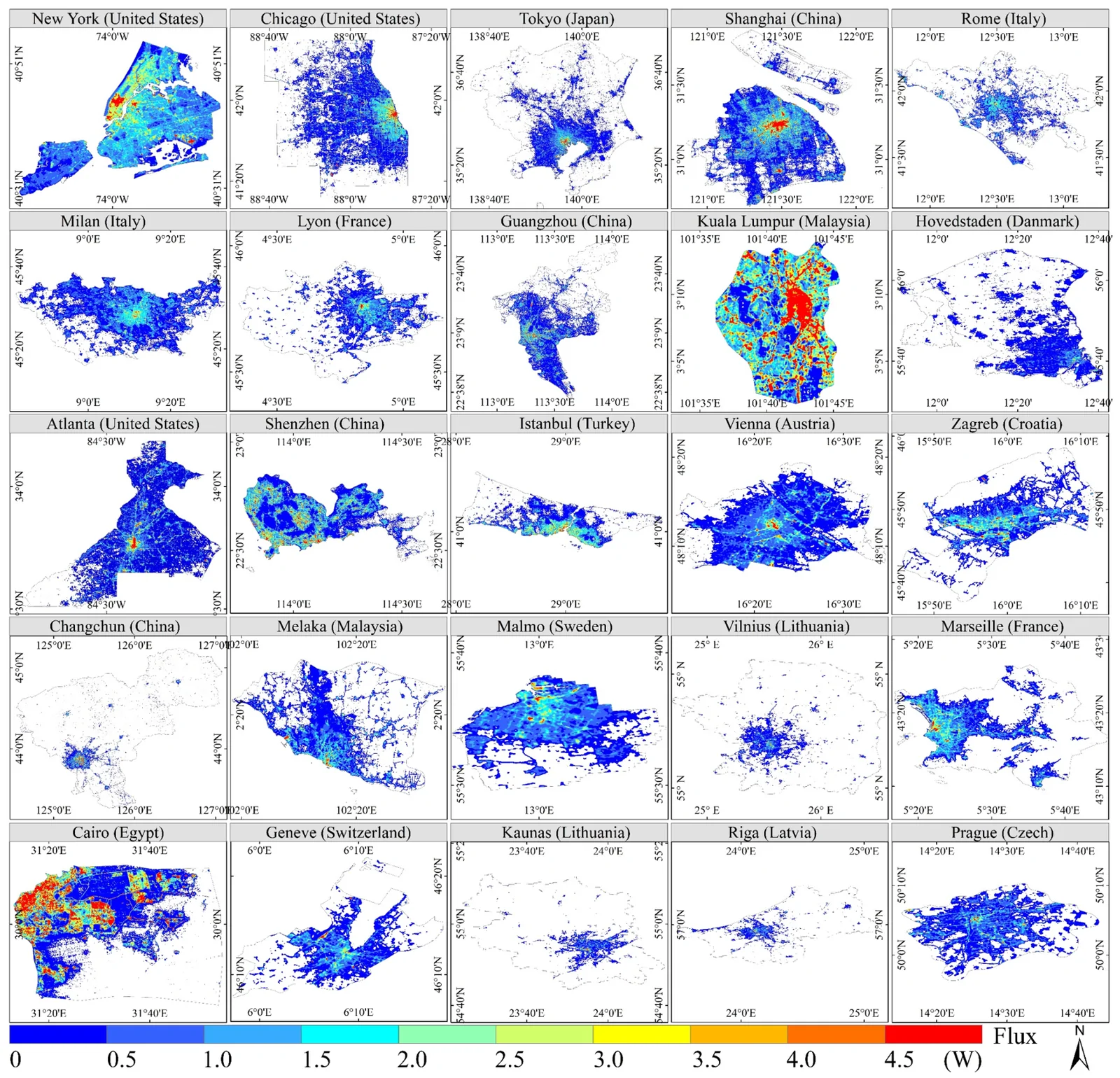
Distribution map of radiance for 25 typical cities in the Northern Hemisphere.

**Figure 7:**
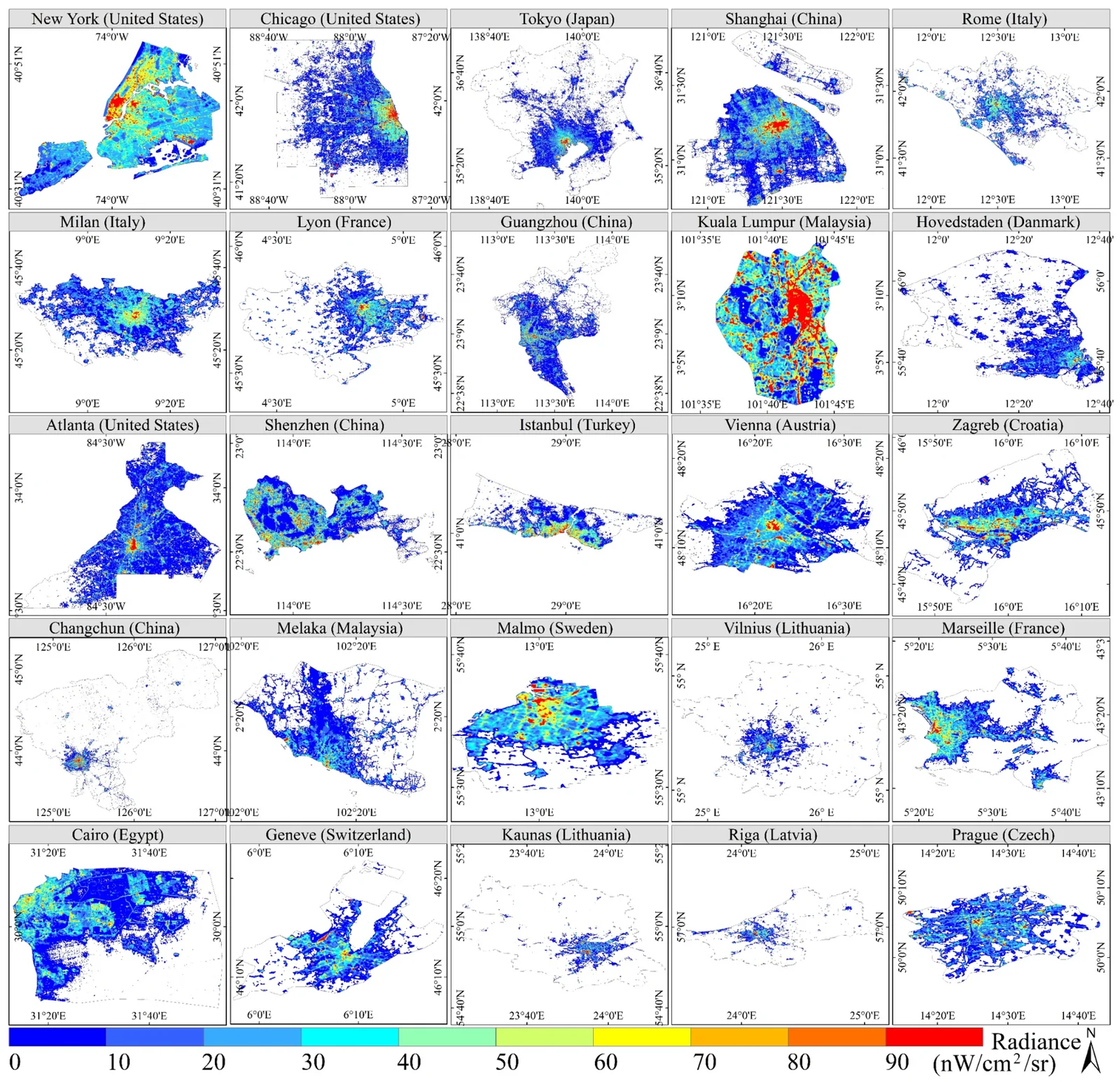
Distribution map of radiant flux for 25 typical cities in the Northern Hemisphere.

**Figure 8:**
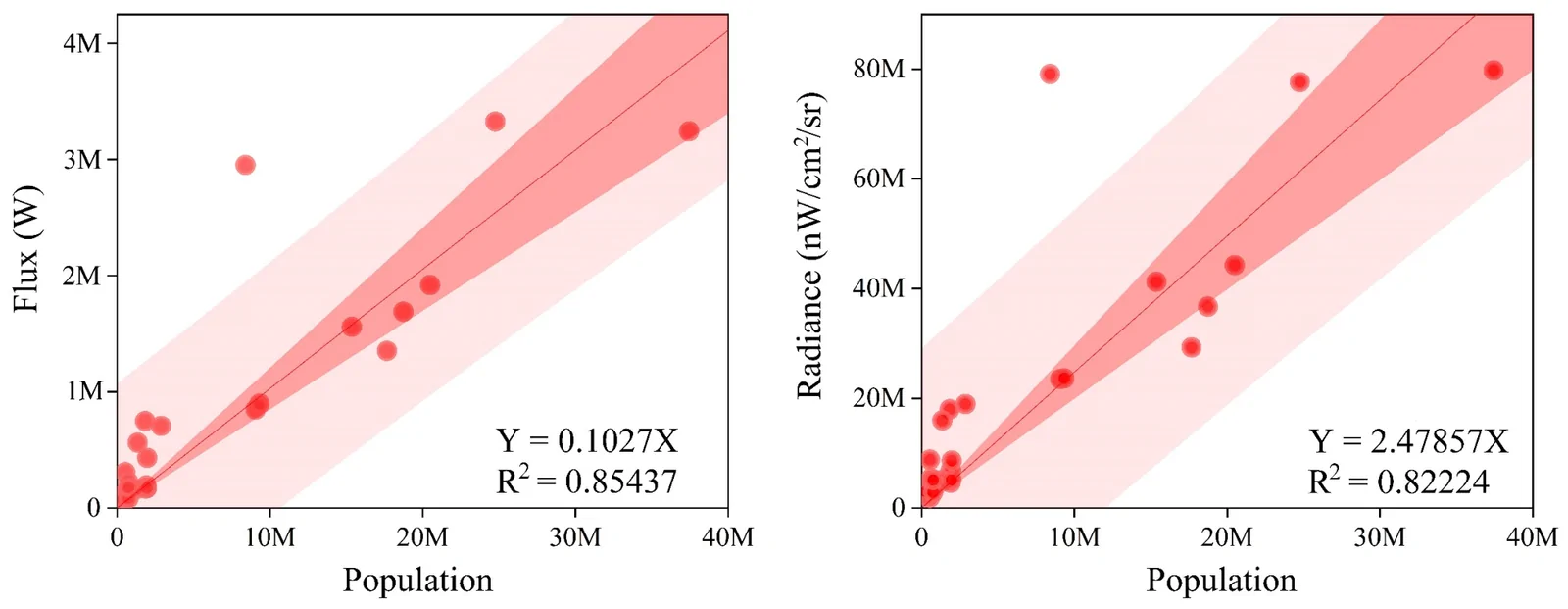
Modeling results of SDGSAT-1 radiant flux and radiance with population.

To evaluate the effect of incorporating grid surface area into regional nighttime light aggregation, this study uses city-level population data as the reference variable. The population data for each administrative unit are sourced from official national statistical agencies, ensuring the authority and consistency of the data. Specifically, we selected 25 representative cities in the Northern Hemisphere as research samples and conducted two regression analyses: one between total radiance and population, and the other between radiant flux and population (Figure 6 and Figure 7). The results show that the goodness of fit (R^2^) between population and radiant flux reached 0.85, improving upon the value of 0.82 obtained using total radiance. In addition, the radiant-flux model achieved a lower RMSE and MAE of 5,273,192 and 2,717,194 respectively, compared with 5,763,507 and 3,552,267 for total radiance, corresponding to reductions of approximately 8.5% and 23.5%. These results suggest that explicit grid-area weighting improves the consistency between aggregated nighttime light measurements and city-level population, particularly when comparing regions located at different latitudes (Figure 8).

### 4.2. Latitude Impact on Radiance and Radiant Flux

**Figure 9:**
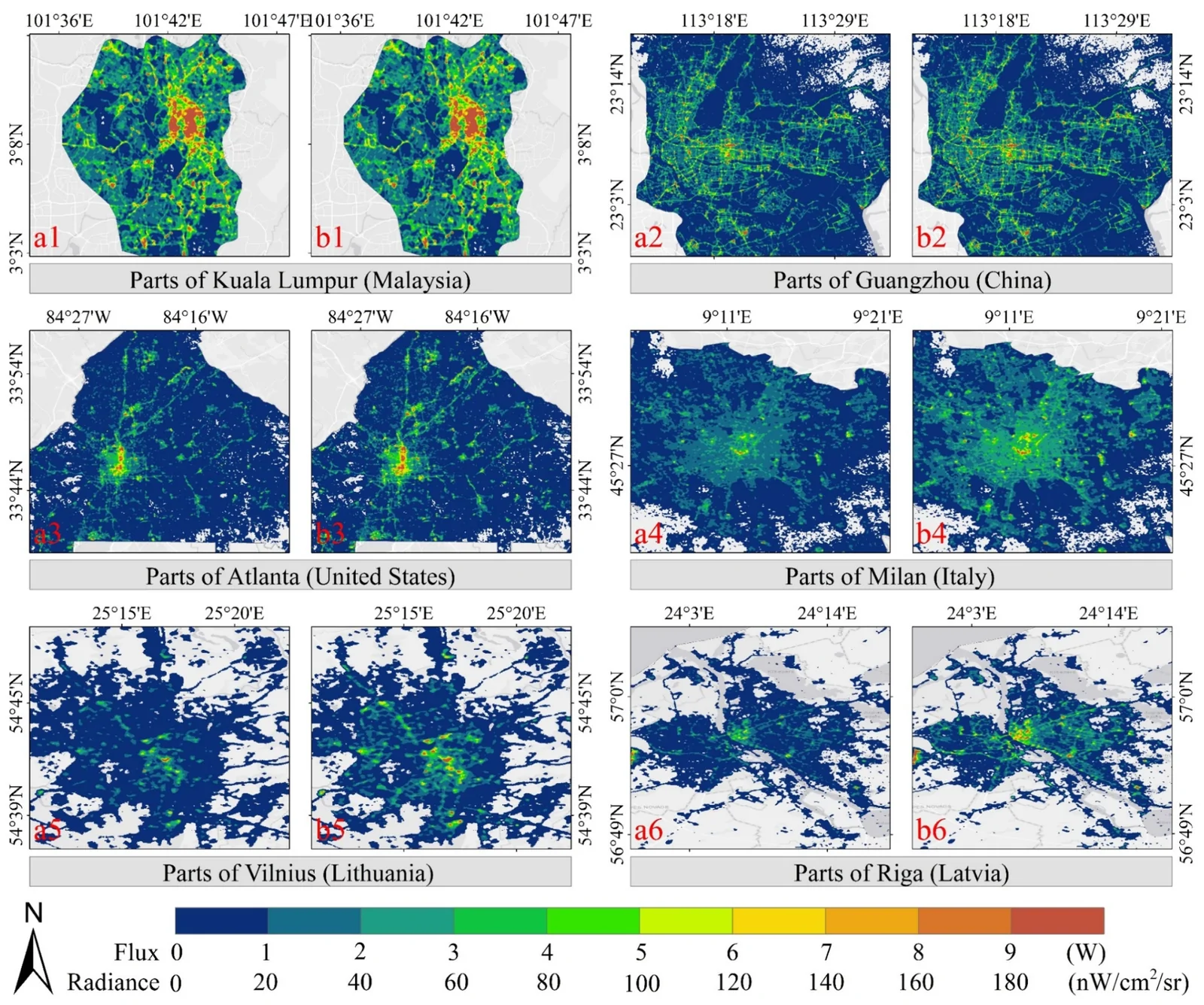
Comparison of the spatial distribution of SDGSAT-1 radiant flux and radiance in six cities located at different latitudes. Panels (**a1**–**a6**) show the spatial distribution of SDGSAT-1 radiant flux, while panels (**b1**–**b6**) show the spatial distribution of SDGSAT-1 radiance. The latitude of the selected cities progressively increases from (**a1**,**b1**) to (**a6**,**b6**).

**Figure 10:**
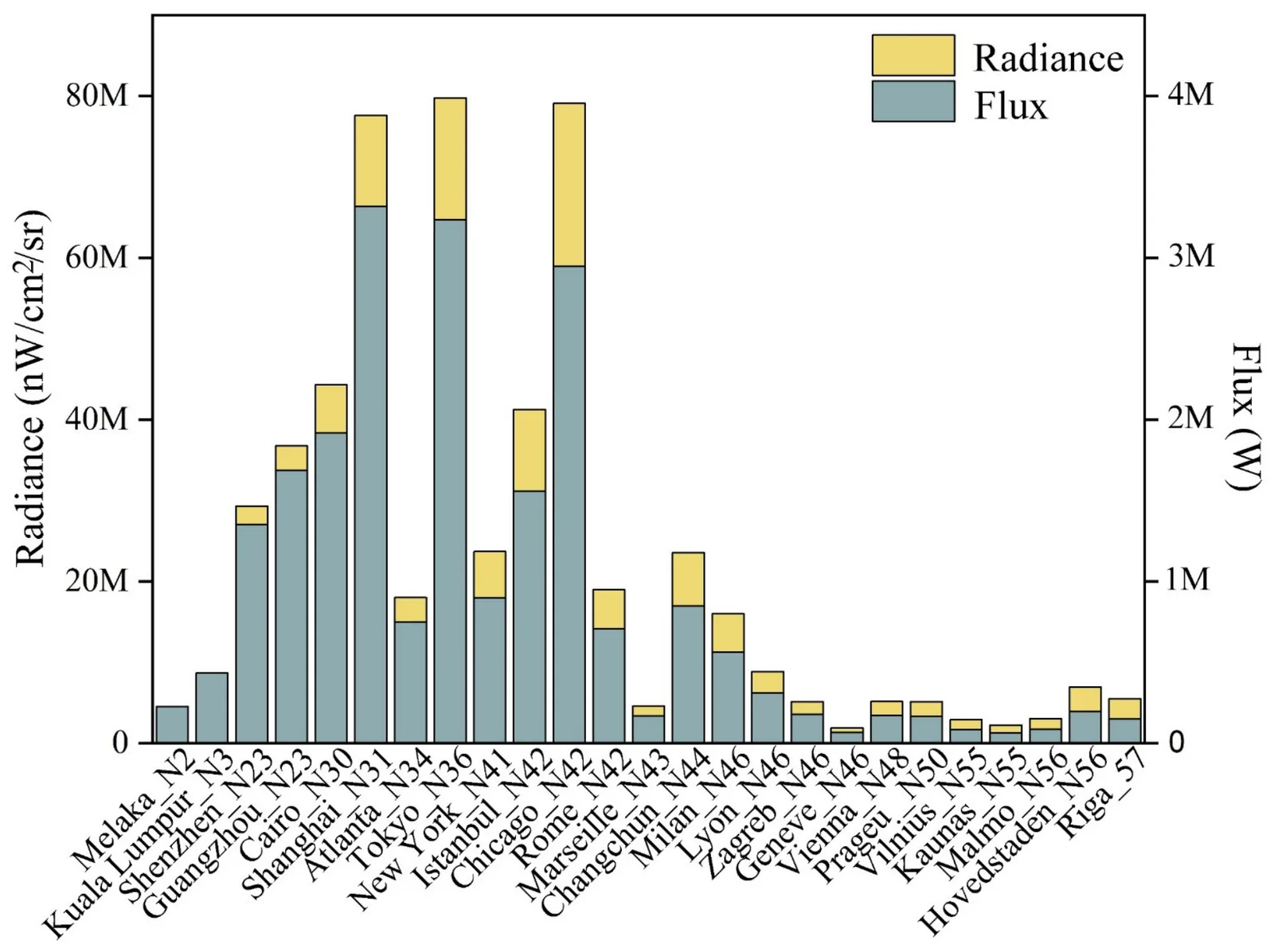
Comparison of the total radiant flux and radiance.

To further explore the spatial distribution differences between radiant flux and radiance, we carefully selected six cities spanning different latitude bands as research samples: Kuala Lumpur (3° N), Guangzhou (23° N), Atlanta (34° N), Milan (46° N), Vienna (48° N), and Riga (57° N). By constructing comparative maps of radiant flux and radiance, we can visually observe the differing trends exhibited by the two variables as latitude changes. Notably, compared to radiance, radiant flux shows a more pronounced attenuation effect as latitude increases (Figure 9). The underlying physical principle behind this finding is that radiant flux, as a physical quantity measuring the radiant energy per unit area per unit time, is directly related to the actual surface area covered by the grid. In other words, the radiant flux value of each grid on the map not only reflects the radiance of that region but also implicitly includes the key factor of grid area. As latitude increases, the curvature of the Earth’s surface causes the actual area corresponding to the same latitude interval to gradually decrease. Therefore, although the radiance in each city remains relatively stable and unaffected by grid area changes, the radiant flux shows a clear diminishing trend due to the reduction in grid area (Figure 10).

**Figure 11:**
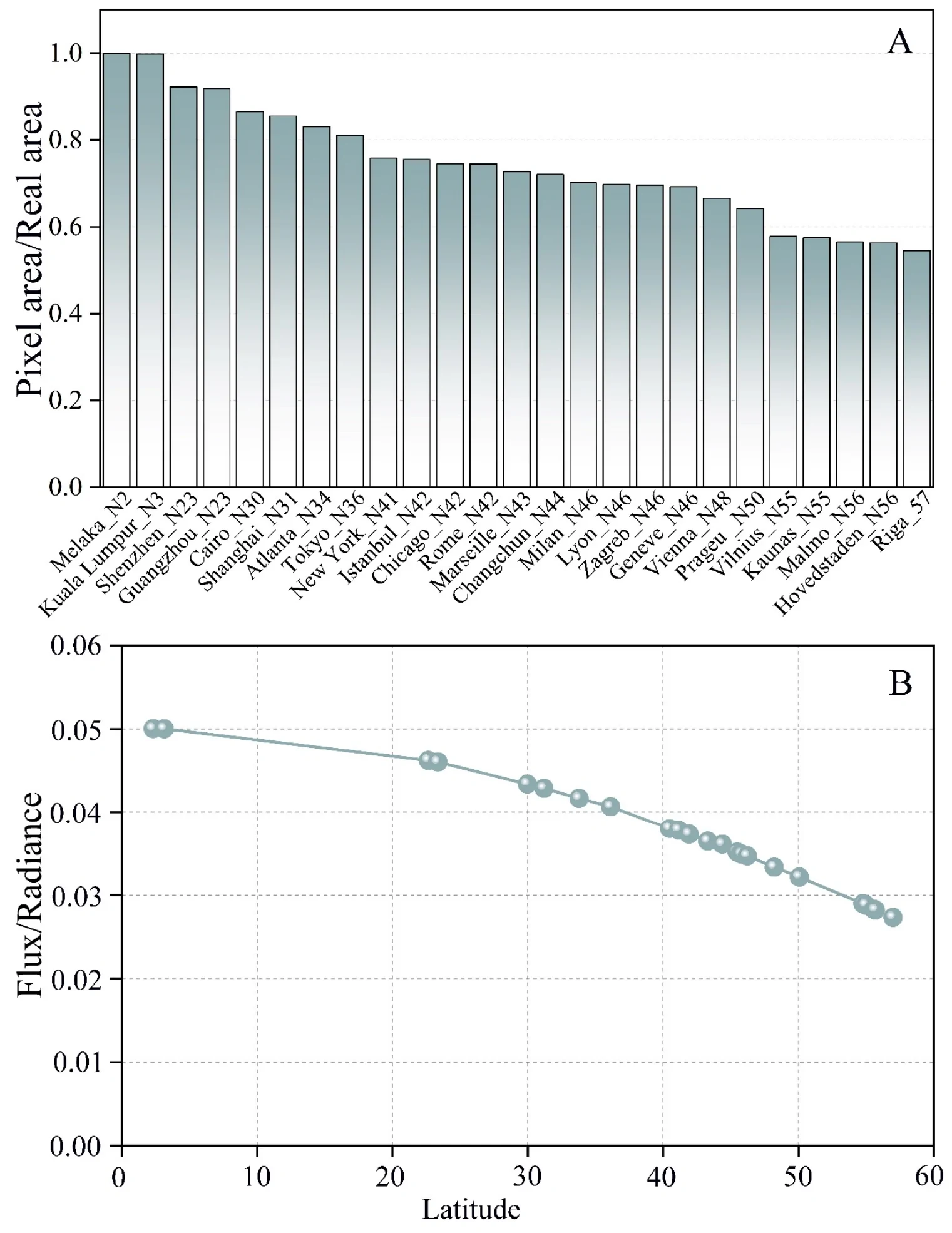
(**A**) Grid area difference between considering and not considering latitude differences (the range is from 0 to 1) for each city, and (**B**) the ratio of radiant flux to radiance (unit: m^2^sr).

To explore the impact of latitude differences on NTL grid area, we conducted a statistical analysis of 25 cities in the Northern Hemisphere. Specifically, we calculated the NTL grid area for these cities under two conditions: considering and not considering latitude differences, and further computed the ratio between the two. This ratio reveals a phenomenon: as latitude increases, the difference in NTL grid area caused by latitude differences becomes more pronounced. At the same time, the ratio of radiant flux to radiance decreases with increasing latitude. In addition, we found that the rate of this difference’s attenuation closely correlates with the cosine function (cos). In other words, in high-latitude regions, such as those near 60° N, if latitude differences are not accounted for, the statistical area is twice the actual area. This undoubtedly leads to significant biases when using NTL radiance to assess population-related indicators (Figure 11).

### 4.3. Comparative Analysis of SDGSAT-1 and VIIRS/DNB

**Figure 12:**
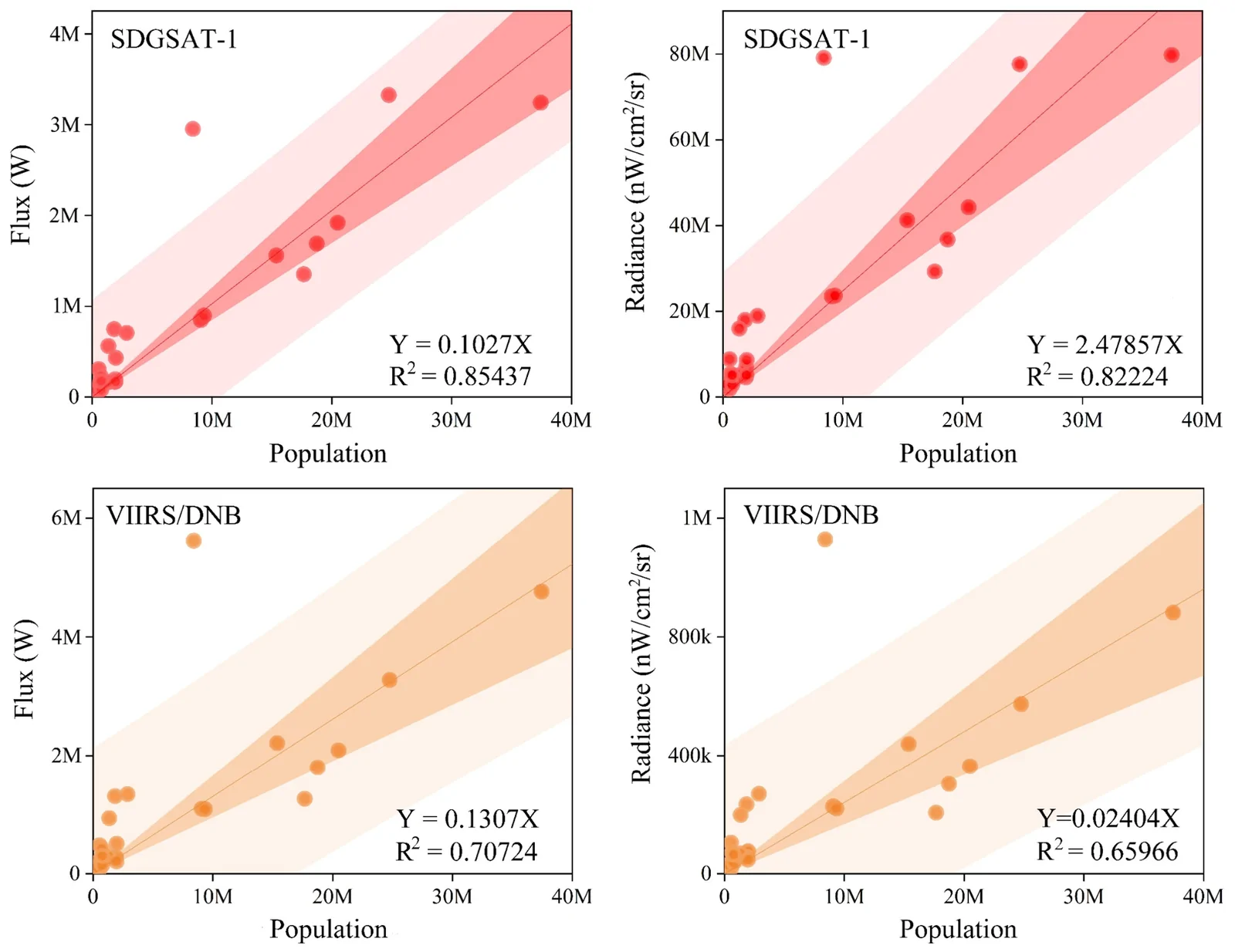
Modeling accuracy of radiant flux and radiance from SDGSAT-1 and VIIRS/DNB data with demographic data.

In addition, this study also constructed regression models based on two indicators, total radiance and radiant flux, derived from VIIRS/DNB datasets, using demographic data at the same city scale, to test the applicability of the proposed radiant flux framework under different sensors. The results show that the radiant flux model exhibits a significantly better fit to demographic data (*R*^2^ = 0.71) compared to the traditional total radiance indicator (*R*^2^ = 0.66) (Figure 12). This improvement trend is highly consistent with the results from SDGSAT-1 data, where radiant flux outperforms total radiance (*R*^2^ = 0.85 vs 0.82). The area-weighted radiant-flux metric showed a stronger relationship with regional population than the unweighted sum of radiance. This result suggests that explicitly incorporating grid surface area may improve population-related regional aggregation across different nighttime light datasets.

**Figure 13:**
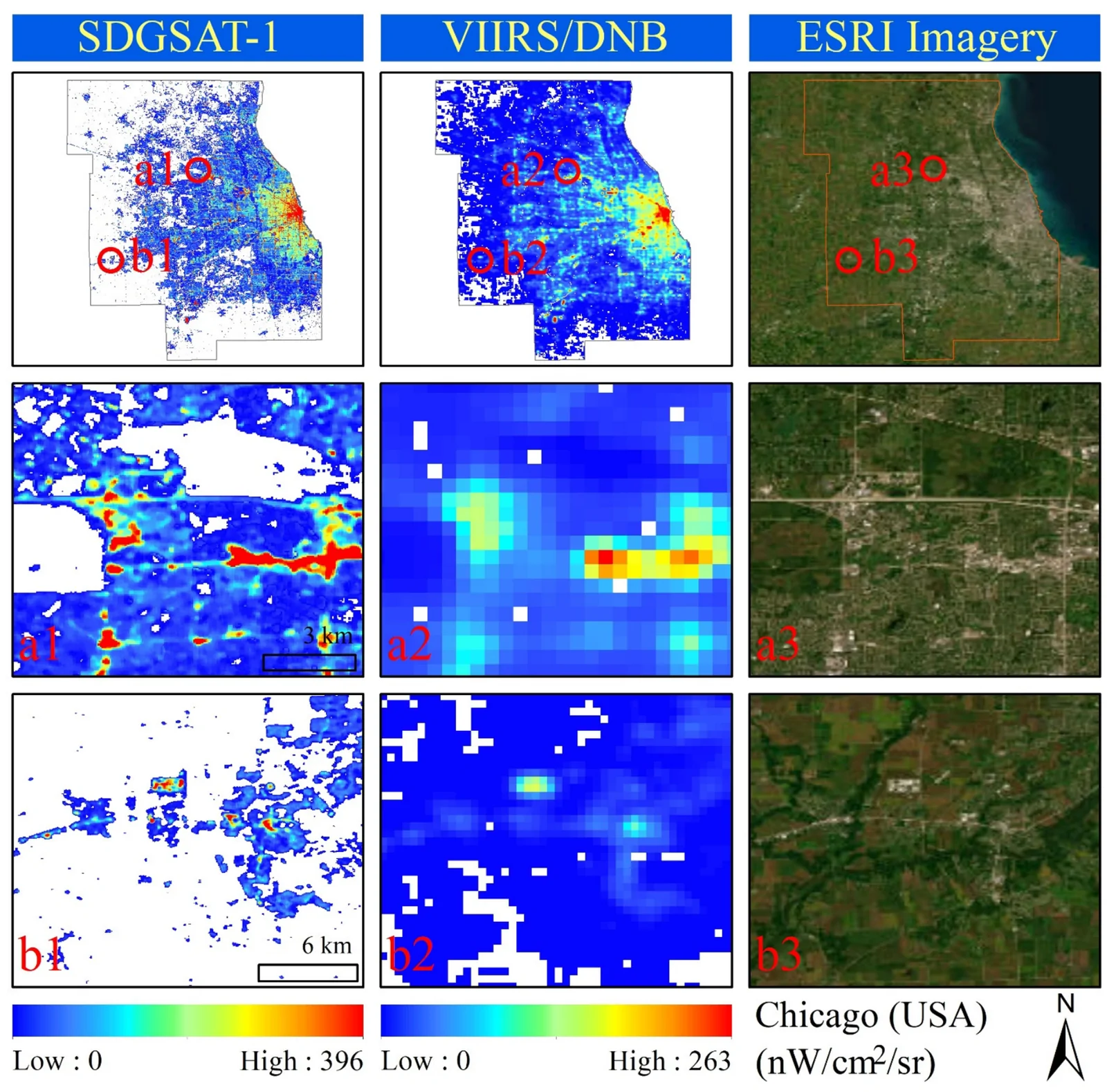
Spatial detail comparison of SDGSAT-1 and VIIRS/DNB NTL data for Chicago, IL, USA. (**a1**–**a3**,**b1**–**b3**) denote two selected areas (a and b) for local spatial comparison, corresponding to SDGSAT-1, VIIRS/DNB, and ESRI Imagery, respectively.

It is worth noting that the VIIRS/DNB data, whether based on radiant flux or total radiance, shows significantly poorer fitting performance with demographic data compared to SDGSAT-1 (Figure 12). To further analyze the reasons behind this difference, we selected Chicago, a representative city in the United States, as a case study for a direct comparison of VIIRS/DNB and SDGSAT NTL data. The comparison results reveal several notable differences between SDGSAT-1 and VIIRS/DNB NTL data. First, SDGSAT-1 NTL has a higher resolution, allowing it to capture finer spatial details, and the hierarchical structure of the urban landscape is clearly presented in SDGSAT-1 NTL images. Second, SDGSAT-1 NTL has made significant progress in addressing light spillover, effectively reducing the edge blurring caused by light scattering, which is crucial for accurately defining the boundaries of human settlement areas (Figure 13). Previous studies have already demonstrated that higher-resolution nighttime light observations can better capture fine-scale urban structures, reduce spatial mixing, and improve the delineation of human settlements [52,53]. Consistent with these established findings, the SDGSAT-1 imagery used in this study preserves more detailed spatial information than VIIRS/DNB, particularly along roads, built-up boundaries, and heterogeneous urban areas. Taken together, these characteristics may collectively contribute to the stronger fitting performance of SDGSAT-1 with population data.

### 4.4. Effect of Image Resolution on NTL Energy Estimation: Radiant Flux vs. Radiance

**Figure 14:**
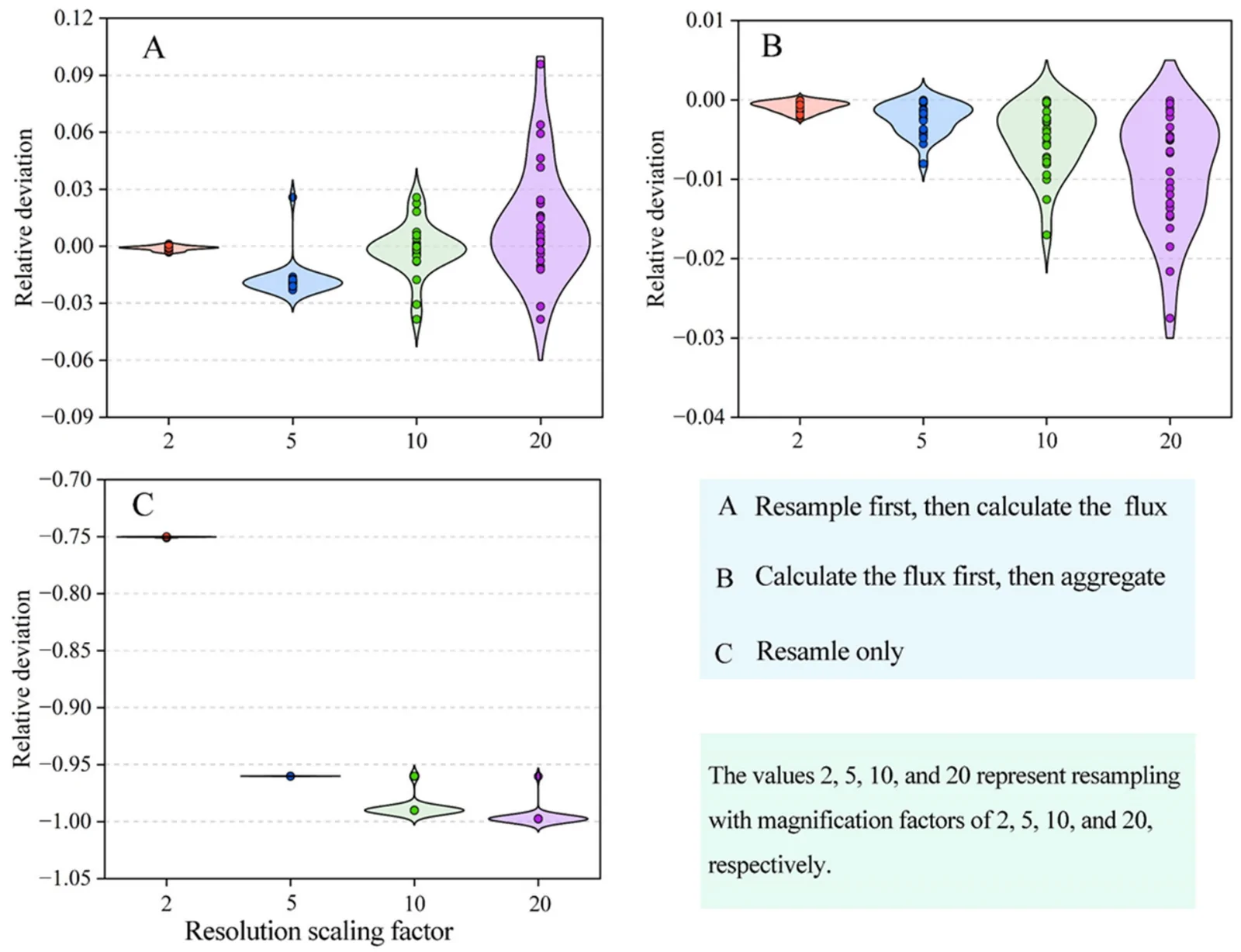
Relative deviation of radiant flux and radiance with resolution changes.

To systematically assess the influence of spatial resolution on NTL radiometric characteristics, the original SDGSAT-1 nighttime light imagery was aggregated to 2×, 5×, 10×, and 20× coarser resolutions, and the corresponding radiant flux and radiance were calculated for 25 representative cities. The results show that regional radiant flux remains relatively stable across the tested aggregation scales, with substantially smaller relative changes than radiance (Figure 14). This pattern reflects the area-integrated nature of radiant flux, which incorporates grid-cell area and therefore preserves regional light totals more effectively during spatial aggregation. Nevertheless, some variation remains across resolutions, indicating that resampling and the loss of fine-scale spatial information can introduce local numerical differences.

In contrast, the summed radiance shows greater sensitivity to spatial aggregation. As spatial resolution becomes coarser, multiple fine-resolution grid cells are combined into fewer and larger grid cells, with their radiance values averaged during the resampling process. Consequently, although the radiance of each coarse grid cell represents the average intensity of the underlying finer cells, the total number of grid cells contributing to the regional summation decreases substantially. Directly summing these averaged radiance values therefore results in a marked decline in the regional summed radiance as spatial resolution decreases. Radiant flux, by explicitly incorporating the actual area represented by each grid cell, is much less affected by this change in grid number and provides a more stable representation of regional nighttime light totals across different spatial aggregation scales.

Further experiments compared two processing sequences: resampling the imagery before calculating radiant flux and calculating radiant flux before spatial aggregation. The results showed a very strong correspondence between the two approaches, with Pearson correlation coefficients exceeding 0.9997 across all tested resolutions. Because correlation alone does not quantify numerical agreement, MAE and RMSE were additionally calculated to characterize the magnitude of the differences between the two approaches (Table 2). The mean absolute relative difference was 0.083%, 1.726%, 0.840%, and 2.721% at the 2×, 5×, 10×, and 20× aggregation scales, respectively. These differences did not follow a strictly monotonic pattern with decreasing spatial resolution, suggesting that the numerical discrepancies may also be influenced by scale-dependent effects associated with resampling, grid alignment, and boundary-cell treatment. Nevertheless, the relative differences remained small overall, indicating that the regional radiant-flux estimates were generally stable under the two processing sequences.

## 5. Discussion

SDGSAT-1 incorporates RGB three-band imaging into satellite-based NTL sensors, with a multispectral resolution of 40 m and a panchromatic resolution of 10 m, offering freely accessible data. Compared to previous single-channel low-light imaging products, it can further reveal spectral structures and spatial gradients, thereby more accurately characterizing human nighttime activities, lighting types, and color temperature differences. This capability gives it unique value in socio-economic indicator assessments, light pollution monitoring, and alignment with goals such as SDG7, SDG11, and SDG15. However, existing studies largely continue to use simple summation of radiance, ignoring significant differences in actual grid area under varying latitude and projection conditions, leading to systematic underestimation of grid cells in high-latitude areas with the same radiance but smaller surface areas. Furthermore, this metric is physically closer to the summation of radiant density, making it difficult to establish a direct, robust dimensional correspondence with total illumination/energy consumption.

Previous studies have recognized the influence of grid-cell area variation in nighttime light aggregation and have addressed this issue through equal-area projection, area-weighted statistics, and latitude correction approaches, particularly for DMSP-OLS and VIIRS/DNB products. However, most previous studies focused on reducing spatial distortion during statistical aggregation rather than transforming radiometric intensity into a physically interpretable area-integrated quantity [53,54,55,56,57]. In this study, we extend this concept by converting gridded SDGSAT-1 radiance into radiant flux through explicit consideration of grid-cell surface area. We propose introducing grid area as a weight factor in the radiance-to-radiant flux conversion model, shifting the analysis from summing radiant intensity to the cumulative estimation of energy flux over unit time. Our results demonstrate that radiant flux offers several key advantages over radiance for cross-latitude comparisons and population-related applications.

First, the comparison between radiance and radiant flux in terms of population estimation highlights the superiority of radiant flux. The goodness of fit between population data and radiant flux (*R*^2^ = 0.85) is significantly better than the fit for radiance (*R*^2^ = 0.82). This result is particularly important for applications where accurate population estimation is crucial, as it ensures that energy assessments are not skewed by the geometric distortions caused by latitude differences. The improved fit for radiant flux in both low-latitude and high-latitude regions demonstrates the robustness of this model in varying geographical contexts. In terms of practical applications, the conversion from radiance to radiant flux has broad implications for various socio-economic and environmental studies. For instance, the improved consistency of radiant flux allows for more reliable assessments of urban development, energy consumption, and light pollution across different geographical regions. This is particularly important for sustainable development monitoring, where policymakers need precise, comparable data to inform decisions related to energy use, urban planning, and environmental protection. Additionally, the comparison between SDGSAT-1 and VIIRS/DNB datasets further underscores the advantages of the radiant flux framework. While both datasets show improvements with radiant flux over radiance, SDGSAT-1 provides superior fitting to population data due to its higher spatial resolution. This highlights the value of SDGSAT-1 in providing fine-grained, accurate information on urban NTL, which is critical for urban planning and disaster management. The ability to capture high-resolution, multi-band data allows SDGSAT-1 to overcome some of the limitations inherent in older sensors like VIIRS/DNB, especially when it comes to light spillover and edge blurring. Finally, the impact of spatial resolution on NTL data was explored by resampling SDGSAT-1 data to various resolutions. Our findings indicate that radiant flux remains stable across different resolutions, providing a robust energy measure even when the spatial detail is lost. This is in contrast to radiance, which shows sensitivity to resolution changes. This makes radiant flux a more reliable indicator for broad-scale studies, where consistent data is needed across different regions with varying resolutions.

It is important to acknowledge the key assumption underlying the radiance-to-radiant-flux conversion proposed in this study. Each grid cell is approximated as a Lambertian surface, allowing radiance to be converted into an area-integrated radiant-flux estimate using the relationship Φ = L × S × π, where L represents radiance and S represents the surface area of each grid cell. This treatment provides a physically interpretable first-order approximation for incorporating spatial area information into an otherwise intensive radiometric quantity. However, actual urban nighttime light emissions are not perfectly Lambertian and may exhibit substantial angular anisotropy, particularly at the 10–40 m spatial resolution of SDGSAT-1. Such anisotropy can arise from the directional emission characteristics of artificial light sources, three-dimensional urban morphology, building obstruction and shadowing, terrain conditions, sensor viewing geometry, surface reflection properties, and atmospheric scattering. More sophisticated angular emission functions have therefore been developed to better represent the spatial distribution of upward light. For example, Falchi et al. (2016) adopted a parameterized angular emission function that extends the simple Lambertian representation and provides a more realistic description of upward light emissions [58].

Given these complexities, the radiant flux derived in this study should not be interpreted as the absolute physical total power emitted by a city into the hemisphere. Rather, it represents a latitude-corrected and area-weighted radiant-flux metric derived under the Lambertian approximation. Future studies could further incorporate more sophisticated angular emission models, where data availability and computational conditions permit, to improve the characterization of the spatial distribution of urban upward light emissions.

It should be noted that the empirical validation in this study focuses primarily on city-level population estimation. Although nighttime light data have been widely used to characterize various socio-economic activities, including economic output, electricity consumption, urbanization, and infrastructure development, these dimensions were not directly examined in the present study. Therefore, the improved performance of the radiant flux metric demonstrated here should currently be interpreted specifically in the context of cross-city population estimation. Future research should incorporate additional socio-economic indicators and independent datasets to further evaluate the applicability, robustness, and transferability of the proposed framework across different socio-economic contexts.

Another important boundary condition of our framework concerns the analytical scale and intended application. The radiant flux metric derived in this study is designed for regional-scale aggregation rather than grid-level analysis. Because radiant flux represents area-integrated light output, it is suitable for applications that require a single comparable measure for a city, province, or other spatial unit, particularly when comparing regions across different latitudes. At the grid scale, however, radiance remains the more appropriate metric because it represents local lighting intensity independently of grid area. Recent studies have also shown that relatively calibrated nighttime light data, or even raw DN values, can be sufficient for applications focusing on within-city spatial patterns, temporal dynamics, or relative comparisons [59,60]. These applications differ from the regional aggregation problem addressed here. When nighttime light values are summed across spatial units at different latitudes, variations in the actual ground area represented by individual grid cells can introduce systematic inconsistencies in regional totals. Therefore, the latitude-related grid-area adjustment proposed in this study is intended specifically to improve the physical consistency and cross-latitude comparability of aggregated nighttime light estimates, rather than to imply that such correction is necessary for all urban nighttime light applications.

In conclusion, this study presents a latitude correction framework for SDGSAT-1 NTL data by shifting the analytical focus from radiance, a density-based measure, to radiant flux, a metric of total emitted power. This approach enhances cross-latitude comparability and provides a physically consistent basis for aggregating nighttime light. The framework contributes to a more robust understanding of NTL data and offers improved analytical support for policy-making in urban development, light pollution mitigation, and sustainability planning.

## 6. Conclusions

This study critically examined the conventional use of summed nighttime light radiance for regional aggregation and identified two key limitations. First, grid area varies systematically with latitude, which introduces bias when total radiance is compared across cities located at different latitudes. Second, the direct summation of radiance, as an intensive and density-based quantity, does not provide a physically explicit representation of total emitted energy. To address these limitations, we developed a physically consistent framework that converts SDGSAT-1 radiance into radiant flux by explicitly incorporating grid surface area. The framework was empirically evaluated using 25 cities in the Northern Hemisphere. The results showed that radiant flux provided a stronger relationship with city-level population than summed radiance, with the coefficient of determination increasing from 0.82 to 0.85. These findings indicate that incorporating grid area can improve the physical interpretability and cross-latitude comparability of aggregated SDGSAT-1 nighttime light data within the Northern Hemisphere. However, the empirical validation in this study was limited to cross-city population estimation. Therefore, the demonstrated improvement should not be generalized directly to other socio-economic or ecological applications. The proposed method provides a new framework for the regional aggregation of SDGSAT-1 nighttime light data, but its effectiveness for GDP estimation, electricity consumption, light pollution exposure, ecological impact assessment, and Southern Hemisphere applications requires further validation using additional indicators, independent datasets, and representative samples from broader geographic and seasonal conditions.

## Figures and Tables

**Table 1:** Wavelengths, gain, and bias parameters for each spectral band.

Band	Wavelength	Gain	Bias
Band_B	424–526 nm	0.0000099253	0.0000099253
Band_G	506–612 nm	0.0000050700	0.0000060840
Band_R	600–894 nm	0.0000135400	0.0000136754

**Table 2:** Comparison of radiant flux estimates obtained using two processing sequences at different aggregation scales.

Resolution Scaling Factor	Pearson r	MAE	RMSE	Mean Absolute Relative Difference
2×	0.9999998	485.98	728.75	0.083%
5×	0.9999976	14,882.85	22,717.10	1.726%
10×	0.9999774	4827.86	8231.54	0.840%
20×	0.9997057	15,005.15	25,286.68	2.721%

## Data Availability

Data will be made available on request.
